# Artificial Intelligence and Sensor Technologies in Dairy Livestock Export: Charting a Digital Transformation

**DOI:** 10.3390/s23167045

**Published:** 2023-08-09

**Authors:** Suresh Neethirajan

**Affiliations:** Department of Animal Science and Aquaculture, Faculty of Computer Science, Dalhousie University, Halifax, NS B3H 4R2, Canada; sneethir@gmail.com

**Keywords:** precision livestock farming, smart agriculture, digital livestock management, animal welfare technology, sustainable livestock production, dairy cow monitoring, IoT in agriculture, agricultural big data

## Abstract

This technical note critically evaluates the transformative potential of Artificial Intelligence (AI) and sensor technologies in the swiftly evolving dairy livestock export industry. We focus on the novel application of the Internet of Things (IoT) in long-distance livestock transportation, particularly in livestock enumeration and identification for precise traceability. Technological advancements in identifying behavioral patterns in ‘shy feeder’ cows and real-time weight monitoring enhance the accuracy of long-haul livestock transportation. These innovations offer benefits such as improved animal welfare standards, reduced supply chain inaccuracies, and increased operational productivity, expanding market access and enhancing global competitiveness. However, these technologies present challenges, including individual animal customization, economic analysis, data security, privacy, technological adaptability, training, stakeholder engagement, and sustainability concerns. These challenges intertwine with broader ethical considerations around animal treatment, data misuse, and the environmental impacts. By providing a strategic framework for successful technology integration, we emphasize the importance of continuous adaptation and learning. This note underscores the potential of AI, IoT, and sensor technologies to shape the future of the dairy livestock export industry, contributing to a more sustainable and efficient global dairy sector.

## 1. Introduction

The dairy sector, a cornerstone of global economies, grapples with challenges such as disease control, animal welfare, and supply chain inefficiencies [1]. If unresolved, these issues could limit productivity and sustainability. The advent of Artificial Intelligence (AI) and advanced sensor technology is instigating paradigm shifts in traditional sectors, including the dairy livestock export industry [2]. These technologies promise to redefine the sector’s landscape [3] by promoting humane, efficient, and sustainable practices.

Exporting dairy livestock involves logistical and welfare challenges. Livestock are exposed to various stresses during long shipping durations, potentially compromising their health. Accurate livestock counting and ensuring there is sufficient feed during transit are major challenges. Given the increasing global demand for dairy products and livestock for breeding, it is imperative to improve livestock export procedures.

Precision digital livestock farming, underpinned by AI and sensor technology, offers innovative solutions to persisting issues in the dairy livestock export industry. These disruptive technologies facilitate real-time monitoring, proactive intervention, and data-driven decision making, promising enhanced animal welfare, productivity, and streamlined supply chain operations [4,5].

**Figure 1:**
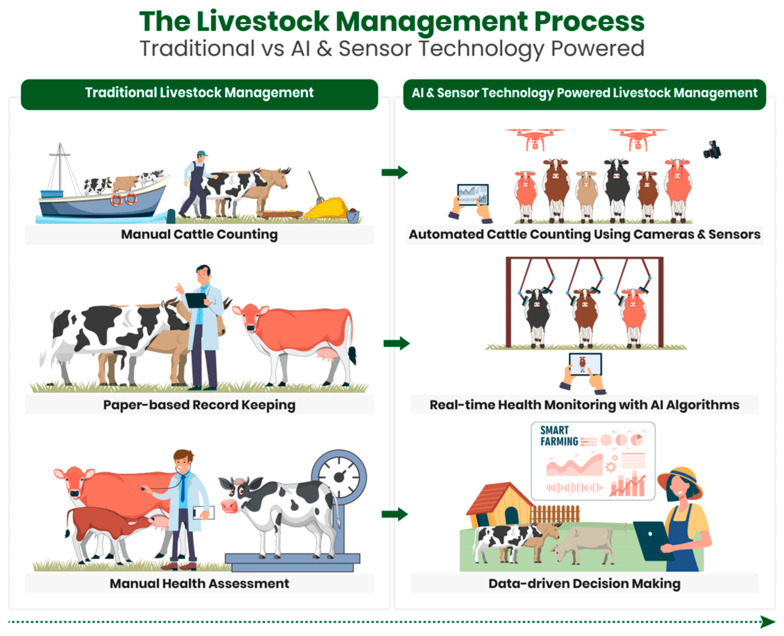
The livestock management process: traditional vs. AI and sensor-technology-powered.

Traditional practices often falter in managing animals (Figure 1), struggling to meet their nutritional needs due to a lack of assertiveness (shy feeders) in reaching feeding troughs in group feeding scenarios [6,7,8]. This behavioral pattern adversely impacts their health and productivity, leading to undernutrition, weight loss, decreased productivity, increased disease susceptibility, and, potentially, a shortened lifespan [9,10]. Current management methods have proven inadequate, underscoring the need for fresh approaches.

Tracking individual animal weights, a crucial parameter of health and performance [11,12], along with accurate cattle counting during the export process [13,14], remains laborious, time consuming, and error prone when using conventional methods. These inaccuracies can lead to supply chain discrepancies, inducing financial losses and logistical complications.

Emerging AI and sensor technology bring forth a beacon of hope [15,16]. By enabling real-time monitoring and data analytics, they can address these concerns, thereby enhancing operational efficiency and animal welfare, crucial elements for the long-term sustainability of the dairy livestock export industry [17].

AI and sensor technology can automate the tracking of individual animal feeding behaviors [18,19], including their time spent near feeding troughs, feeding frequency, and intake [20]. These data, once analyzed using AI algorithms, can facilitate early interventions. Furthermore, these technologies assist in monitoring feeding behaviors—vital for livestock management—as they provide insights into animals’ health, productivity, and overall wellbeing. Systems typically employing RFID tags or smart collars can automate this process, offering real-time data [20]. These systems track proximity to feeding troughs, feeding frequency [21], and duration. Analyzing these data can alert farmers of anomalies, enabling early health issue detection [22,23], and aid in optimizing feed management, thereby enhancing productivity and sustainability.

This technical note aims to contribute to the evolution of a more sustainable, efficient, and humane dairy livestock export industry through a focused exploration of the Internet of Things (IoT), sensor, and Artificial Intelligence (AI) technologies. Central to our investigation are three key applications: managing feeding behaviors, automating livestock weight tracking, and improving cattle counting accuracy during transportation and traceability.

We delve into the broader implications of these technologies within the dairy livestock export sector. Our analysis encompasses the potential for advancements in animal welfare, operational efficiency, and market access and competitiveness. Concurrently, we address the challenges emerging from the adoption of these cutting-edge technologies, including data security, privacy, infrastructure demands, sensor data reliability, interpretability of AI insights, ethical considerations, and cost implications.

Subsequently, this note outlines a strategic roadmap for the seamless incorporation of these technologies, offering insights into the future trajectory of the sector. Ultimately, this note seeks to serve as a valuable resource for stakeholders within the dairy livestock export industry, enabling informed decision making and fostering innovation.

## 2. Application of AI and Sensor Technology in Livestock Management

**Figure 2:**
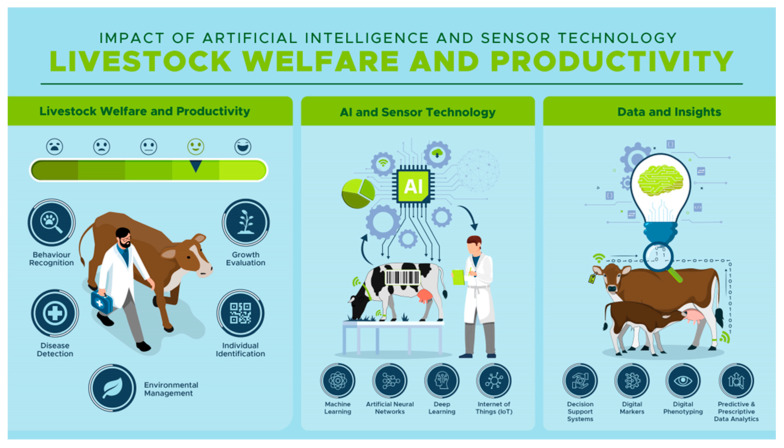
Impact of AI and sensor technology on livestock welfare and productivity.

Artificial Intelligence (AI) and sensor technologies are propelling industries into the future, and the dairy livestock export industry is no exception. As a sector steeped in tradition, this industry is beginning to feel the transformative impact of these advanced technologies (Figure 2). AI and sensor technologies are not only changing the way farmers and exporters operate but also shaping the future trajectory of the industry.

In any livestock population, certain animals stand out due to their lack of assertiveness in reaching feeding troughs, especially in group feeding scenarios. These animals, colloquially referred to as ‘shy feeders’, exhibit a lower food intake [15]. This behavior significantly impacts their health and productivity, often leading to undernutrition, weight loss, decreased productivity, a heightened susceptibility to diseases, and, in some instances, a reduced lifespan [16,17]. Shy feeder cows, those that eat less when in a group, can be at risk during the shipping process as they may not get enough nutrition.

Given these repercussions, the identification and effective management of shy feeders become pivotal. Historically, this issue has been addressed through manual observation and intervention, which is both time consuming and labor intensive, and not always accurate or timely. However, with the advent of AI and sensor technology, the management of shy feeders is poised for a significant shift. AI can be used to analyze behavioral patterns of livestock through video and motion sensors. This can help in identifying shy feeder cows and modifying the feeding strategies accordingly.

The role of monitoring feeding via AI and sensor technologies offers innovative solutions (Table 1) to the challenges in livestock management, including identifying and managing animals with feeding difficulties [20]. These technologies automate the tracking of individual animal feeding behaviors, including their time spent near feeding troughs, feeding frequency, and intake. These data, when analyzed using AI algorithms, can assist in identifying animals with feeding difficulties, thereby facilitating early interventions [20,21].

In addition, the role of monitoring feeding behaviors in livestock management provides invaluable insights into animals’ health, productivity, and overall wellbeing. AI and sensor-based systems revolutionize this aspect by offering real-time, automated monitoring capabilities [20]. These systems employ technologies such as RFID tags or smart collars to track various parameters, converting raw data into meaningful insights about the animals’ feeding behaviors [22,23]. By addressing long-standing challenges such as the optimization of feeding behavior, these technologies can significantly enhance animal welfare, streamline supply chain operations, and boost the dairy livestock export industry’s productivity and profitability [24,25].

Within the sphere of livestock management, the weight of an animal serves as a vital health and productivity barometer. Traditional methods of weight collection in livestock farming rely heavily on manual weighing, a labor-intensive, time-consuming, and error-prone process. AI and sensor technology emerge as game changers in this landscape, fostering an environment for automated data capture in livestock farming [25,26].

The use of AI and sensor technology provides revolutionary solutions (Table 2) for streamlining operations in the livestock supply chain, from counting and tracking animals to predicting and optimizing the logistical routes [27,28]. RFID technology offers transformative potential in the field of automated cattle counting, a critical factor in disease control, inventory management, animal movement tracking, productivity enhancement, and livestock enterprise profitability [27,29,30]. Moreover, sensor technologies and AI algorithms, through real-time monitoring and predictive analytics, provide vital insights into every stage of the supply chain. Sensors tracking feed as well as water consumption, for instance, can signal potential health risks, prompting AI algorithms to forecast possible disease outbreaks and recommend preventative measures [31,32].

AI and sensor technology also extend their impact to market access and development in the livestock export industry. The traceability offered by RFID and AI technology can meet the stringent safety and quality standards of import markets, bolstering a country’s access to these markets [33]. Furthermore, AI can analyze sensor-generated data from various supply chain stages to discern valuable insights about market trends, consumer preferences, and price fluctuations, aiding strategic planning and market expansion efforts.

Overall, AI and sensor technologies offer boundless opportunities for operational efficiency, animal welfare enhancement, traceability assurance, and market access expansion in the livestock export industry. The onus now lies on livestock enterprises, policymakers, and researchers to foster an environment conducive to harnessing the potential of these technologies, bringing us closer to a future where every element of the livestock supply chain operates as a cog in a well-oiled machine, powered by AI and sensor technology.

## 3. Future Perspectives: AI and Sensor Technology in Livestock Management

### 3.1. Identifying Opportunities and Overcoming Challenges

As we delve into the future of livestock management especially from a practical industry perspective, it is evident that the role of Artificial Intelligence (AI) and sensor technology is increasingly vital. Cutting-edge innovations such as sophisticated machine learning algorithms, expansive big data analytics, widespread Internet of Things (IoT) connectivity, and drone technology herald a new era in the realm of livestock farming.

Machine learning, for instance, has the potential to fine tune predictive analytics, delivering even more accurate and timely insights into animal health, productivity, and potential supply chain issues. The proliferation of IoT devices could facilitate an expansive real-time tracking and monitoring system for livestock, bringing critical data to the fingertips of farmers and industry stakeholders.

**Figure 3:**
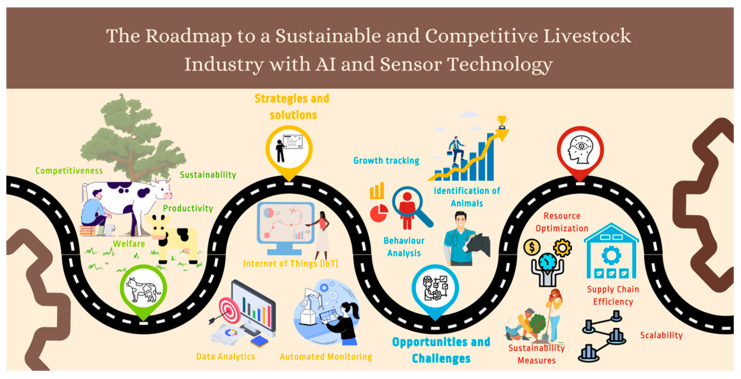
The roadmap to a sustainable and competitive livestock industry with AI and sensor technology.

Nonetheless, these advancements come hand in hand with substantial challenges (Figure 3). A key concern is the enormous volume of data generated by these technologies. While the depth of big data is invaluable, it necessitates substantial storage, processing capabilities, and advanced analytics tools to convert it into actionable intelligence [34]. Further, integrating these sophisticated technologies into current livestock management systems can prove complex, disruptive, and capital intensive, necessitating significant technical know-how.

The issue of data privacy and security also looms large. With vast amounts of data collected and shared, there are legitimate apprehensions regarding the security and potential misuse of this information [35]. A heavy reliance on technology simultaneously heightens the risk of cyber-attacks, with potential repercussions for the entire livestock management system.

These challenges call for a well-rounded, strategic response [36]. Technological solutions such as robust data encryption, cloud storage, and improved analytical tools can help manage and secure the data avalanche. Concurrently, there is a pressing need to establish comprehensive regulatory frameworks to safeguard privacy rights and prevent data misuse.

### 3.2. Navigating towards a Sustainable and Competitive Livestock Sector

AI and sensor technology represent pivotal tools for steering the livestock industry towards a more sustainable and competitive future. By optimizing resource use, boosting productivity, and improving animal welfare, these technologies can catalyze a livestock industry that is economically robust, environmentally benign, and socially responsible.

For example, precise monitoring of animal health can mitigate the reliance on antibiotics, addressing a critical environmental and public health issue. Similarly, the efficient management of feed and water resources not only curtails costs but also minimizes waste and environmental degradation. Further, enhanced supply chain efficiency can strengthen competitiveness by reducing losses, elevating product quality, and ensuring punctual delivery. As the global demand for livestock products escalates, these efficiencies could provide a decisive advantage in the highly competitive international market.

Yet, realizing this vision necessitates a collaborative effort from all stakeholders. It requires a continuous investment in technology development and deployment, supportive policies, and robust public–private partnerships. Above all, it mandates a paradigm shift: technology must be perceived not merely as an instrument for augmenting productivity but as an indispensable tool for achieving sustainability and resilience.

### 3.3. Applied Informatics for Dairy Livestock Export: Overcoming Big Data Challenges for Real-Time Analytics and Sustainable Practices

In the swiftly transforming world of dairy livestock export, the quest for proficient analytics capable of a dynamic and automated processing of the temporal–spatial distribution of animals in real time has surfaced as a crucial necessity. This necessity stems from the increasing demand for efficiency, transparency, and sustainability in the livestock industry, driven by both market forces and societal expectations. The capacity to precisely track and forecast the movement and behavior of livestock in real time can dramatically augment operational efficiency, animal welfare, and overall productivity. It can also provide valuable insights into the health and well-being of the animals, thereby contributing to improved animal welfare standards and more sustainable farming practices.

However, the road to achieving this level of sophistication in livestock management analytics is laden with hurdles. The most formidable of these challenges are scalability and robustness. The enormous volume of data generated in real time from a myriad of sources, including GPS trackers, RFID tags, machine vision system cameras, and IoT sensors, can be daunting. This ‘big data’ scenario calls for scalable solutions that can efficiently process and analyze data on a colossal scale without compromising speed or accuracy.

The challenge of scalability is not just about handling large volumes of data, but also about integrating and making sense of diverse types of data. For instance, GPS data can provide information about the location and movement of animals, while data from IoT sensors can provide insights into their health status and environmental conditions. Integrating these diverse data sources and extracting meaningful insights from them requires sophisticated data processing and analytics capabilities.

Furthermore, the analytics solutions must exhibit robustness to deliver reliable predictions and insights in real time, under fluctuating conditions and potential system anomalies. This requires not only robust algorithms but also robust data infrastructure and data management practices. For instance, data quality issues, such as missing or erroneous data, can significantly impact the accuracy and reliability of analytics results. Therefore, robust data cleaning and data quality management practices are essential.

Traditional statistical techniques and machine learning approaches, such as decision trees or random forests, have been utilized in the past to analyze livestock data. While these methods have their merits, they may fall short in handling the complexity and scale of real-time, big data scenarios in the livestock export industry. These techniques often grapple with high-dimensional data and may not provide the level of accuracy required for real-time decision making.

In light of these limitations, there is a burgeoning consensus in the agrifood domain that advanced machine learning and deep learning approaches could be the panacea to these challenges. Deep learning, a subset of machine learning inspired by the structure and function of the human brain, has demonstrated remarkable success in handling high-dimensional data and delivering accurate predictions in various fields, including image recognition, natural language processing, and autonomous vehicles.

In the context of dairy livestock export, deep learning models could be trained to recognize patterns and make predictions based on a multitude of factors, including the spatial–temporal distribution of animals, their health status, environmental conditions, and market trends. These models could potentially provide more accurate and timely insights, enabling farmers and exporters to make better-informed decisions, optimize their operations, and ultimately, enhance the sustainability and profitability of their enterprises. However, the adoption of deep learning in the agrifood sector is not without its challenges. These include the need for large amounts of labeled training data, the complexity of model development and tuning, and the interpretability of model predictions. The need for large amounts of labeled training data can be particularly challenging, as it requires significant time and effort to collect and label data. Moreover, the complexity of model development and tuning requires specialized skills and expertise, which may not be readily available in the agrifood sector.

Moreover, the successful integration of deep learning models into the livestock management workflow would require significant investment in infrastructure, skills development, and change management. This includes not only the physical infrastructure for data storage and processing but also the software infrastructure for data management, model development, and deployment. Skills development is another critical aspect, as it requires the training and upskilling of staff to effectively use and manage the advanced analytics solutions. Change management, on the other hand, involves managing the organizational changes associated with the adoption of new technologies and practices.

In addition to these challenges, the interpretability of model predictions is another critical issue. Deep learning models, often referred to as ‘black boxes’, can make highly accurate predictions but may not provide clear explanations for their predictions. This lack of interpretability can be a significant barrier to the adoption of deep learning in the agrifood sector, where decision makers often need to understand the reasons behind the predictions to make informed decisions. Despite these challenges, the potential benefits of adopting advanced analytics and deep learning in dairy livestock export are immense. These benefits extend beyond improved operational efficiency and productivity to include enhanced animal welfare, more sustainable farming practices, and increased competitiveness in the global market. By providing real-time, accurate, and actionable insights, advanced analytics can enable farmers and exporters to make better-informed decisions, optimize their operations, and respond more effectively to market trends and changes.

Moreover, by improving the tracking and monitoring of animal health and welfare, advanced analytics can contribute to higher standards of animal welfare and more ethical farming practices. This, in turn, can enhance the reputation and brand value of dairy livestock exporters, making them more attractive to consumers and investors who value sustainability and animal welfare.

While the path towards advanced analytics in dairy livestock export is challenging, the potential benefits in terms of improved efficiency, animal welfare, and profitability make it a journey worth undertaking. As researchers, developers, and industry stakeholders continue to explore and innovate in this space, the future of dairy livestock export looks set to be increasingly data driven, intelligent, and sustainable. The journey towards this future will require not only technological innovation but also a collaboration and partnership among various stakeholders, including farmers, exporters, technology providers, researchers, and policymakers. By working together, these stakeholders can overcome the challenges and unlock the full potential of advanced analytics in dairy livestock export.

## 4. The Intersection of Sensor Technologies and Artificial Intelligence: A Closer Look

Sensor technologies and AI form a critical intersection in modern livestock management. Sensors provide a way to collect real-time data from livestock. The vast amounts of data collected can be overwhelming and seemingly chaotic, which is where AI steps in, decoding these massive data sets, identifying patterns, and providing actionable insights [37].

### 4.1. Sensor Technologies

Sensor technologies can be categorized into two types: wearable devices and environment-based sensors. Wearable sensors are devices attached directly to the animal. They may track physiological parameters (e.g., heart rate, body temperature), behavioral traits (e.g., feeding patterns, movement), and other relevant indicators of an animal’s health and welfare [38].

Environment-based sensors, on the other hand, monitor the conditions around the animals. These could include video cameras, thermal imaging sensors, accelerometers, load cells in feeding stations, and drones, among others. They can provide a wealth of information about the environment and how animals interact with it [39].

The application of sensor technologies in livestock management has opened new avenues for the in-depth monitoring of animals in ways that were previously impossible. However, there are challenges such as the durability of wearable devices, potential discomfort or injury to the animal, ensuring the devices stay on the animals, and the cost and complexity of installing and maintaining environment-based sensors [40].

### 4.2. Artificial Intelligence

AI is a broad field that encompasses machine learning, deep learning, computer vision, and more. It provides the capability to analyze and interpret the massive data sets collected by sensors. AI’s ability to ‘learn’ from data and make predictions makes it a powerful tool for decoding the vast array of livestock data [41,42].

In the context of dairy livestock export, AI can be used for a variety of applications. It can identify patterns in livestock behavior and physiological parameters to detect illness, stress, or discomfort. It can analyze patterns in feeding behavior to identify shy feeders and adjust the feeding strategies. It can recognize and count individual animals in video footage, and it can use data on feeding behaviors and physiological parameters to optimize the feeding schedules and portions. However, AI also poses challenges. Developing accurate AI algorithms requires substantial amounts of high-quality training data. There are also ethical considerations associated with AI, such as privacy concerns and the potential for the misuse of data [43].

### 4.3. Integration of Multiple Sensor Modalities

One of the future directions in this area is the integration of multiple sensor modalities for the comprehensive monitoring of animal health. A single sensor can only provide a limited perspective. For instance, a wearable device may monitor heart rate, but it might not be able to provide insights into the environmental factors influencing the animal’s stress levels. By integrating data from wearable sensors, environmental sensors, and video data, a more holistic understanding of the animal’s condition can be obtained [44].

### 4.4. Advancements in AI Algorithms

Advancements in AI algorithms will also play a significant role in the future of dairy livestock export. Current AI models, such as machine learning and deep learning algorithms, are already powerful tools for analyzing livestock data. However, these models could be further improved. For instance, developing algorithms that can analyze multiple types of data (e.g., physiological data, environmental data, video data) simultaneously could provide more comprehensive and accurate insights [45].

### 4.5. Customization and Individual Animal Approach

As with any technology application, one size does not fit all. Dairy cattle have individual differences in their behavior, physiology, and response to environmental stressors. These differences need to be taken into account when designing and implementing sensor and AI systems. For example, the optimal position and type of wearable sensor might vary depending on the size, breed, and behavior of the cow. AI algorithms also need to be designed to account for the individual differences between cows [46,47].

### 4.6. Technological Adaptation and Training

The implementation of sensor technologies and AI systems in the dairy livestock export sector requires adequate training for staff. Staff must be trained to install and maintain the technologies, interpret the data generated, and take the appropriate actions based on the insights provided by AI. Additionally, the dairy cattle must adapt to the new technologies, particularly the wearable devices. Proper training and adaptation are crucial for the successful implementation of these technologies [48].

### 4.7. Stakeholder Engagement and Consumer Perception

The use of sensor technologies and AI in dairy livestock export has implications beyond the farm gate. Stakeholders, including consumers, have increasingly high expectations for animal welfare, environmental sustainability, and food safety. The use of these technologies can help meet these expectations by improving animal welfare and reducing the environmental impacts. However, there is also a need to effectively communicate with stakeholders about the use of these technologies to avoid misconceptions and ensure that they are accepted [49].

### 4.8. Data Security and Privacy

With the rise of sensor technologies and AI, vast amounts of data are being collected and analyzed. This presents significant challenges in terms of data security and privacy. Ensuring the secure storage and transmission of data is crucial to prevent unauthorized access and the misuse of data. Regulations and best practices need to be developed and implemented to ensure data security and privacy [50,51,52].

### 4.9. Economic Considerations

While sensor technologies and AI offer many benefits, they also come with costs. The initial investment in the hardware, software, and training can be substantial. There are also ongoing costs for maintenance and data management. Therefore, careful economic analysis is necessary to ensure the benefits outweigh the costs. This includes not only the direct economic benefits but also indirect benefits such as improved animal welfare, reduced environmental impacts, and enhanced public perception [53,54,55].

### 4.10. Ensuring Animal Comfort and Welfare

While wearable devices offer valuable data on an individual animal’s health and well-being, it is vital to ensure that these devices do not compromise the comfort or welfare of the animals. Sensor devices should be designed and fitted in a way that minimizes the potential discomfort, injury, or stress for the animals. Regular checks are needed to ensure the devices remain in the correct position and are not causing any harm to the animals. It is also essential to consider the potential stress associated with introducing new technologies and to manage this process carefully to minimize stress for the animals [56,57,58].

### 4.11. Technology Integration and Interoperability

Given the variety of sensor technologies and AI applications that can be used in dairy livestock export, there is a need to ensure these technologies can be integrated and can operate together seamlessly. This includes not only the integration of different types of sensor data but also the interoperability of different AI algorithms. Developing standardized protocols for data collection, storage, and analysis can help ensure the smooth integration and interoperability of these technologies [59,60,61].

### 4.12. Continual Monitoring and Evaluation

Finally, as sensor technologies and AI are implemented in the dairy livestock export process, it is crucial to continually monitor and evaluate their effectiveness. This includes not only tracking their performance in terms of improving animal health and welfare, but also assessing their impact on operational efficiency, economic outcomes, and environmental sustainability. Regular evaluations can help identify any issues or areas for improvement and ensure that the technologies are providing the maximum number of benefits [62,63,64,65]. 

Despite significant advances in sensor and Artificial Intelligence (AI) technologies, their adoption within the livestock export sector remains in the early stages. Numerous studies have been conducted to explore the potential of these technologies in enhancing the efficiency and safety of livestock export, but their translation from research and development to practical implementation is still a work in progress.

There are several reasons for this lag in adoption. One of the main challenges is the practical application of technology. Many technologies that function well in controlled lab conditions might struggle in the dynamic and complex real-world scenarios of livestock export. Livestock export operations present varying environmental conditions, which could significantly impact the performance of sensors and AI tools.

Economic factors also play a significant role. The cost of integrating new technology in these operations can be high, and many businesses may hesitate to make such investments unless they are sure of the economic benefits. Technologies that require substantial changes in operational routines or significant capital investments could be especially challenging to implement.

Regulatory hurdles may also slow the adoption of new technologies. Depending on the country and the specific regulations of the livestock industry, gaining approval for the use of certain technologies can be a lengthy and complex process.

Furthermore, resistance from end users can also be a barrier. Established practices are often hard to change, and new technologies that disrupt these practices may not be readily accepted. In such cases, proper training and education about the benefits and usage of these new technologies are crucial for their successful integration.

While there is no shortage of promising research on the use of sensor and AI technologies in livestock export, several challenges still need to be addressed for these technologies to be widely adopted and effectively employed in real-life situations.

### 4.13. Challenges Associated with the Use of Sensors and IoT

Data security and privacy: The use of sensors and AI can lead to the collection of a vast amount of data. How these data are stored, processed, and protected can be a significant concern.

Infrastructure requirements: Implementing AI and sensor technology requires significant infrastructure, including data storage and processing facilities, high-speed internet connections, the expertise of highly qualified personnel, and power sources.

Accuracy of sensors: Ensuring that sensors accurately and consistently record data is vital. Inaccurate or inconsistent data can lead to misinformed decisions and subsequent consequences. Malfunctioning sensors could lead to incorrect decisions based on flawed data.

AI interpretation: While AI can process and analyze large volumes of data far more quickly than humans, interpreting that data in a meaningful and useful way can be challenging. An over-reliance on AI without proper understanding could lead to erroneous decisions.

Ethical considerations: There could be ethical concerns around the use of sensor and AI technology in dairy livestock export, particularly if it is perceived as intrusive or causing stress to the animals.

Cost: The cost of implementing and maintaining advanced technologies such as AI and sensors can be high.

We acknowledge the importance of providing comprehensive details on the particular algorithms, including their precision, constraints, and the dependability of related sensor technologies. Nevertheless, it is crucial to emphasize that a significant proportion of these technologies, while developed in academic environments, have yet to be examined or validated in a commercial context within the livestock industry, specifically in the export division.

## 5. Conclusions

Artificial Intelligence and sensor technology are pioneering a transformational shift in the livestock export industry. These technologies offer innovative solutions to enduring challenges, promising a revolution in livestock management, specifically within the dairy sector. From the automated management of ‘shy feeders’, accurate weight tracking, to efficient cattle enumeration, AI and sensor technologies have the potential to augment productivity, promote animal welfare, and streamline supply chain operations. Yet, this transformational journey is not without significant barriers. The technical complexity, privacy issues, and substantial requirements for capital and expertise present serious challenges that need to be overcome to fully harness these technologies. It thus necessitates a paradigm shift within the livestock export industry from traditional practices to a data-driven, automated operation model. However, the potential impact of these technologies is not limited to operational improvements alone. They also offer the potential for a more sustainable, competitive livestock industry, marrying economic growth with environmental preservation and animal welfare. They envision a future where livestock farming evolves beyond merely being a source of food production to an exemplar of efficiency, sustainability, and humane animal management. As we progress, maintaining a focus on technological innovation, advocating for supportive policy measures, and encouraging robust stakeholder collaborations are key. This review underscores the exciting reality that we are at the brink of a technological revolution in livestock management. The challenge now lies in embracing this change and stepping into a future where AI and sensor technology are integral components of the livestock export industry.

## Figures and Tables

**Table 1:** Key applications of Artificial Intelligence and sensor technology in livestock management.

Application	AI and Sensor Technology Role	Specific Technology Used	Benefits	Limitations
Identification of ‘Shy Feeders’	Uses AI and video analytics to spot ‘shy feeder’ behavior through RFID tag data analysis.	RFID tags, computer vision, machine learning algorithms	Aids early identification and intervention, improving herd health and productivity. Enables personalized nutrition plans.	Needs sensor setup and careful AI calibration to minimize false results.
Monitoring of Feeding Behaviors	Sensors track feeding metrics with AI identifying abnormal patterns in real time, offering actionable insights.	Feed intake sensors, IoT (Internet of Things) connectivity, cloud computing, machine learning algorithms	Gives real-time insights into animal health and nutrition status, enabling timely interventions. Helps prevent over/underfeeding.	Requires robust connectivity and sensor maintenance for real-time monitoring.
Automation of Weight Collection	Sensor-based walk-over-weighing systems with AI interpretation for automatic weight collection.	Walk-over-weighing systems, IoT connectivity, cloud computing, machine learning algorithms	Provides accurate, hassle-free weight tracking. Allows continuous monitoring of animal performance. Assists in adjusting feeding strategies.	Requires animal training to use the system, sensor calibration and maintenance for accurate readings.

**Table 2:** Impact of AI and sensor technology on supply chain management in livestock industry.

Aspect	AI and Sensor Technology Role	Specific Technology	Benefits	Challenges
Automated Cattle Counting	Facilitates accurate, efficient cattle counting using AI-powered image processing.	Machine vision systems, image recognition algorithms	Minimizes human errors, accelerates counting, allows real-time livestock tracking.	Setup needs for cameras and processing systems, varying accuracy due to lighting and cattle movement.
Supply Chain Traceability	Uses sensors for location and condition tracking throughout the supply chain, coupled with AI for real-time tracking and issue prediction.	GPS trackers, RFID tags, IoT connectivity, Big Data Analytics	Boosts traceability, promotes animal welfare through timely interventions, assists in regulatory compliance.	Demands robust sensor network and data management, potential privacy concerns with location tracking.
Market Development	Leverages AI for market trend analysis, demand-supply dynamics, and price fluctuations, offering predictive insights for production and exports.	Machine Learning algorithms, big data analytics	Promotes proactive decision making, optimizes market demand fulfilment, potentially increases profits.	Relies on comprehensive market data, requires advanced AI models for accurate predictions.
